# Kinematics of the Hip and Body Center of Mass in Front Crawl

**DOI:** 10.2478/v10078-012-0040-6

**Published:** 2012-07-04

**Authors:** Ricardo J. Fernandes, João Ribeiro, Pedro Figueiredo, Ludovic Seifert, João Paulo Vilas-Boas

**Affiliations:** 1 Centre of Research, Education, Innovation and Intervention in Sport, Faculty of Sport, University of Porto, Porto, Portugal.; 2 Porto Biomechanics Laboratory, University of Porto, Porto, Portugal.; 3 University of Rouen, Faculty of Sport Sciences, France.

**Keywords:** Swimming, biomechanics, displacement, velocity, applicability

## Abstract

The kinematic profiles of the hip and center of mass in front crawl swimming were compared to quantify the error of using a fixed body point to assess intracyclic velocity variations at moderate intensity exercise. The practical goal was to provide a useful tool, easy and fast to assess, and to use as feedback, for assessing swimming efficiency. Sixteen swimmers performed an intermittent incremental protocol that allowed assessing the individual anaerobic threshold velocity. One complete stroke cycle was analysed at the step intensity corresponding to each swimmer’s anaerobic threshold. The subjects were videotaped in the sagittal plane using a double camera set-up for two-dimensional kinematical analyses. The hip and the center of mass presented similar mean velocity and displacement values, being highly related to both parameters. However, the hip reflects the center of mass forward velocity and horizontal displacement with 7.54% and 3.24% associated error, respectively. Differences between hip and center of mass were observed for intracyclic velocity variations (0.19±0.05 and 0.25±0.08, respectively, for a p<0.001), and the negative mean error value found (−0.06) evidenced a tendency of the hip to overestimate the center of mass velocity variation. It is possible to conclude that the hips forward movements might provide a good estimate of the swimmer’s horizontal velocity and displacement that is relevant for diagnostic purposes, especially to assess swimming efficiency through the intracyclic velocity variations. Nevertheless, the hip point error magnitude should be taken into consideration in data interpretation.

## Introduction

Evaluation of swimmers is an essential tool for increasing the efficiency of the training processes and to predict performance (Smith et al., 2002). From the complex group of factors influencing swimming performance, the biomechanical parameters seem fundamental. Recently, Barbosa et al. (2010) evidenced the importance of the swimmer’s energetic profile and this one from the biomechanical behaviour. In fact, the importance of improving technique to enhance swimming performance is a topic of great interest for coaches and researchers, being observed that 40% of the 662 papers published in the Biomechanics and Medicine in Swimming books (a series of international symposia organized every four years since 1970) had a biomechanical approach (Vilas-Boas et al., 2010).

Studies focusing on swimming biomechanics usually include a kinematic, kinetic, electromyographic or coordinative approach (Barbosa et al., 2008; Schnitzler et al., 2010), but, due to its complexity, swimming technique has been frequently characterized using a simple analysis of the stroking parameters (velocity, stroke rate and stroke length). Its assessment has been carried out since the 1970s (Psycharakis and Sanders, 2009), with long tradition in the technical and scientific swimming community once swimmer’s velocity may be explained by the product of frequency and distance per stroke. However, the increasing recognition of its limitations has led to the development of biomechanical equipment and analytical methods, and to a more frequent quantification of other kinematic parameters related to swimming performance (Holmér, 1979; Alberty et al., 2005).

One well-known parameter for the analysis of technical proficiency (Holmér, 1979; Craig et al., 2006; Tella et al., 2008; Seifert et al., 2010), swimming efficiency (Alberty et al., 2005), motor coordination (Schnitzler et al., 2010), and comparison between swimming intensities (Barbosa et al., 2008) and techniques (Maglischo et al., 1987; Craig et al., 2006) has been the intracyclic velocity variations (IVV). IVV represents the accelerations and decelerations of a swimmer’s fixed body point (or body center of mass, CM) within a stroke cycle. Two methods are frequently used for its assessment: (i) the velocity of a fixed point, mostly the hip, using mechanical or image-based methods (Maglischo et al., 1987; Craig et al., 2006; Schnitzler et al., 2010); and (ii) the 2D and 3D reconstruction of the CM motion through digitizing procedures (Maglischo et al., 1987; Barbosa et al., 2008; Psycharakis et al., 2010).

The assessment of the hip’s IVV using mechanical procedures takes multiple cycles into consideration, being more training relevant once results and outputs are immediate. This procedure is also very simple and less time consuming (Vilas-Boas et al., 2010) and seems more adequate for practical purposes (Schnitzler et al., 2010). Mechanical assessment of IVV may be performed using velocimetry (Costill et al., 1987; Craig et al., 2006; Schnitzler et al., 2010) and accelerometry (Holmér, 1979; Tella et al., 2008), but, when velocimetry is used only a single swimming pool length can be analysed (due to cable impairments), and its validity has been questioned (Psycharakis and Sanders, 2009). If accelerometers are used, this problem is solved, but the outputs interpretation is not so intuitive, requiring time integration to obtain velocity to time functions; in fact, it was already evidenced that the trunk rotations and inertial effects might influence hip motion when no propulsion or resistance is generated (Psycharakis and Sanders, 2009). The digitizing methods, if validated previously, can also be used to determine the IVV of the hip. This analysis has been done mainly in the horizontal axis of motion (Maglischo et al., 1987; Seifert et al., 2010), once 3D assessment is affected by some errors due to the reconstruction procedure (Figueiredo et al., 2009). The CM reconstruction method seems to be more valid (Barbosa et al., 2003; Psycharakis and Sanders, 2009; Psycharakis et al., 2010), but it is very time-consuming (Maglischo et al., 1987), dependent of the precision of the anthropometric biomechanical model used to calculate the inter-limb inertial effects (Schnitzler et al., 2010), and incurs in significant errors from digitizing procedures, distortion and underwater video techniques (Barbosa et al., 2008; Figueiredo et al., 2009). In addition, as only one arm cycle is usually analysed, the inter-cycles variability is not considered, having lower applicability for technical training purposes.

The aim of this study was to compare the 2D kinematic profiles of the hip and CM, considering displacement and forward velocity, to quantify the error magnitude of using a fixed body point to assess IVV when swimming front crawl at moderate intensity, which is one of the metabolic zones most stressed in swimming practice (Olbrecht, 2000). It was hypothesized that the 2D estimation of IVV from the hip accurately represents the IVV of the CM, presenting a relevant practical tool to characterize swimmers technique. However, the use of the hip will imply an associated error with a magnitude that should be known and taken into consideration in data interpretation.

## Material and Methods

Sixteen long distance swimmers voluntary participated in the present study. The mean ± SD values regarding their main physical and training background characteristics are as follows: 29.2 ± 10.3 years, body height : 175.1 ± 4.8 cm, arm span : 176.8 ± 5.1 cm, body mass : 67.7 ± 5.7 kg, body fat : 10.9 ± 6.5 kg, lean body mass : 59.1 ± 5.5 kg, and long distance swimming experience : 6.6 ± 5.9 years. All subjects were involved in at least 5 swimming training sessions per week, and participated in regional and national level competitions. The participants provided informed written consent before data collection, which was approved by local ethics committee and performed according to the declaration of Helsinki.

The test session took place in a 25 m indoor pool, 1.90 m deep, with a water temperature of 27.5º C, during the preparatory phase of the winter macrocyle. A standardized warm-up of 1000 m of low to moderate aerobic swimming intensity was conducted. Briefly, during the morning (from 9 to 12 am), each subject performed a 7 × 200m front crawl individualized intermittent protocol with increments of 0.05 m/s each step (controlled through a visual pacer - TAR. 1.1, GBK-electronics, Aveiro, Portugal), and 30 s rest intervals (Fernandes et al., 2008). Each subject swam alone in one lane, avoiding pacing or drafting effects. Capillary blood samples for blood lactate analysis were collected from the earlobe at rest, during the 30 s rest intervals, at the end of exercise, and during the recovery period (Lactate Pro, Arkay, Inc., Kyoto, Japan), to assess the individual anaerobic threshold through the lactate concentration/velocity curve modelling method (Fernandes et al., 2008; 2010). Swimmers were advised not to get involved in high intensity training 24 h prior to the experiment, and to maintain their daily nutritional habits.

Swimmers were videotaped in the sagittal plane for 2D kinematical analyses using a double camera set-up, with both cameras (Sony^®^ DCRHC42E, 1/250 digital shutter, Nagoya, Japan) fixed on a specially designed support for video image recording placed at the lateral wall of the pool and 12.5 m from the start wall (Barbosa et al., 2008). One camera was placed 0.30 m above the water surface and the other was kept 0.30 m underwater (Sony^®^ SPK-HCB waterproof box, Tokyo, Japan) exactly below the surface camera, and at 6.78 m from the plane of movement. Video images were synchronized in real time using a pair of lights visible in the field of each video camera. Subjects were monitored when passing through a specific pre-calibrated space using bi-dimensional rigid calibration structure (6.30 m^2^) with nine control points.

One complete arm stroke cycle (without breathing) was analysed, being chosen a cycle performed at the middle of the pool (clean swimming) during the 175 m lap of the 200 m step corresponding (or closest) to individual anaerobic threshold velocity. The video images were digitized with the Ariel Performance Analysis System (Ariel Dynamics, San Diego, USA) at a frequency of 50 Hz. The CM reconstruction was obtained using the Zatsiorsky and Seluyanov’s model, adapted by de Leva (1996), which considered 21 anatomical reference points (vertex, 7th cervical, mandible (mental protuberance), humeral heads, ulnohumeral joints, radiocarpal joints, 3rd dactylions, trochanter major of femurs, tibiofemoral joints, talocrural joints, calcanei and acropodion), the Direct Linear Transformation algorithm (Abdel-Aziz and Karara, 1971), and a low pass digital filter of 5 Hz. Considering that the kinematical analysis of the swimming locomotion imposes obstacles to data acquisition, particularly by the existence of errors associated to image distortion, digitisation and 2D reconstruction, it seems important to observe its influence on the final results, analysing validity, reliability, and accuracy (Figueiredo et al., 2011). The reliability of the digitizing procedure was assessed by the intraclass correlation coefficient (ICC) of two consecutive digitisations of a randomly selected trial, being 0.99 and 0.69 (p < 0.001) for displacement and velocity, respectively.

The displacement and forward velocity of the right hip (trochanter major) and CM in the horizontal axis were selected for analysis. The IVV was calculated through the coefficient of variation of the velocity to time mean values (CV = SD/mean), as proposed by Barbosa et al. (2006) and Schnitzler et al. (2010). The maximum and minimum velocities (v_max_ and v_min_, respectively) were also obtained from the instantaneous velocity data. In addition, the relative v_max_ and v_min_ were calculated as a percentage of the stroke cycle mean velocity, and its timing of appearance were computed as a percentage of the stroke cycle duration.

Data were checked for normal distribution with the Shapiro-Wilk test. Considering the CM values as the criterion, the mean error, the root mean square (RMS) error, and the percentage error (RMS error expressed as a percentage of averaged CM values) were calculated for the hip variables. A paired sample t-test and the ICC were used to investigate the relationship between the hip and the CM; the mean ICC was obtained by Fisher’s Z’ transformation. All data were analysed using the SPSS version 17.0 (SPSS Inc., Chicago, USA) and the significance level was set at 5%.

## Results

Table 1 presents the values regarding the mean ± SD of the CM and hip for the forward velocity and displacement in the horizontal motion axis, as well as the mean error, the RMS error, the percentage error and the mean ICC between CM and hip. The CM and hip presented similar values both for velocity and displacement. In fact, the ∼0 values of the mean error indicate that the hip does not under or overestimates the CM velocity values.

However, concerning the displacement variable, a slight tendency for a hip underestimation is shown.

Conversely, concerning the values of RMS error and percentage of error, the hip reflects with higher error the CM in the velocity than in the displacement variable. Furthermore, high positive correlation coefficient values were found between the hip point and the CM regarding both horizontal swimming velocity and displacement.

**Figure 1:**
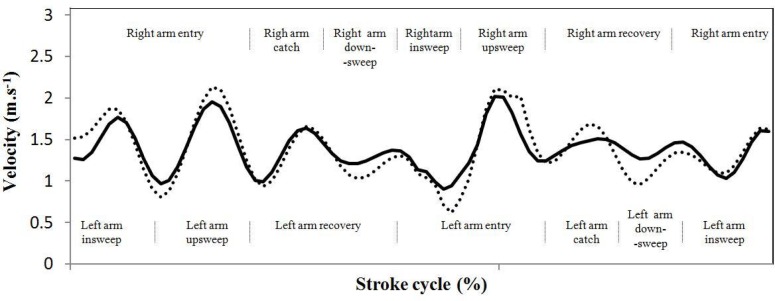
Example of the intracyclic velocity variations of the hip (dashed line) and of the centre of mass (continuous line) for one swimmer

Complementarily, a typical forward velocity to time profile of the hip and CM (for both right and left arm strokes) is displayed in Figure 1, being observable positive accelerations of the hip and CM during the insweep and upsweep phases of the left arm (coincident with the entry of the right arm), and during the catch of the right arm. The hip and CM negative accelerations occurred during the transition between propulsive phases, and in the downsweep coincident with the recovery of the opposite arm. It is also evidenced that the hip presents higher forward velocity peaks magnitude comparing to the CM.

Table 2 presents the descriptive statistics for the CM and hip velocity related variables, showing also the p value regarding eventual differences between CM and hip. The mean and RMS errors are also displayed, evidencing the validity of the hip values when using the CM values as criterion. Differences between CM and hip were observed for IVV, v_max_, v_min_, relative v_max_, and relative v_min_. The negative mean error values found for the IVV, v_max_, relative v_max_, timing v_max_ and timing v_min_ show a tendency of the hip to overestimate the CM values (the positive mean errors illustrate the opposite behaviour). The greater RMS values were identified in the timing of appearance of v_max_ and v_min_ during the stroke cycle.

## Discussion

The key to success in swimming does not rely on hard, but purposeful and careful training (Olbrecht, 2000), meaning that it should be well planned and monitored (Smith et al., 2002). Knowing that changes of the horizontal velocity during a stroke cycle is a topic increasingly popular among coaches and researchers (Psycharakis and Sanders, 2009; Barbosa et al., 2010; Vilas-Boas et al., 2010), the objective of this study was to compare the IVV kinematic profiles of the hip and CM in front crawl swimming to quantify the error of using a fixed body point to assess IVV. As IVV is an important indicator of swimming technique (Barbosa et al., 2008), which is a major factor influencing swimming performance (Costill et al., 1987; Smith et al., 2002). The pertinence of the current study is perfectly justified once it has great practical application.

The above-referred analysis was conducted at an intensity corresponding to the metabolic individual anaerobic threshold velocity, i.e., at the highest exercise intensity at which a balance between the production and removal of lactate occurs (Olbrecht, 2000). This velocity was selected since it is often used in training, representing the maximum aerobic velocity that swimmers can maintain without accumulation of fatigue (approximately 30 min) (Olbrecht, 2000; Fernandes et al., 2010). Previous studies conducted in order to observe whether the hip accurately represents the intracycle CM profile in front crawl have been carried out at much higher intensities (Maglischo et al., 1987; Psycharakis and Sanders, 2009). As results, higher IVV values were expected due to a significant increase in both propulsive and drag forces (Schnitzler et al., 2010). In fact, Barbosa et al. (2006) found a linear relationship between IVV and energy cost, and, therefore, with velocity, in the front crawl.

In the current study, a 2D kinematical recording was implemented since it requires less digitizing time and has fewer methodological problems. In fact, the 2D approach is conceptually easier to relate to, and can yield acceptable results (Bartlett, 2007), being proper to evaluate numerous samples and to implement in field studies, particularly in the swimming club. Conversely, the 3D analysis is a very time-consuming process that requires complex analytical methods, what makes it difficult for coaches to use on a day-to-day basis (Psycharakis and Sanders, 2009).

CM and hip presented similar mean values for both forward velocity and displacement. Such a result was expected once the CM is located in the hip region (Costill et al., 1987; Maglischo et al., 1987; Figueiredo et al., 2009). In fact, nonetheless the mean error concerning the hip and CM displacement towards a slight tendency for a hip underestimation, the approximately 0 velocity mean error values indicate that the hip seems not to under or overestimate the CM velocity values. This is in line with the literature, as Maglischo et al. (1987) concluded that forward velocity of the hip can be a useful tool for diagnosing problems within stroke cycles. However, the values of RMS error and percentage of error evidence the opposite behaviour: although being of low magnitude, the error is higher regarding forward velocity (7.54%) than the displacement (3.24%). It is accepted that the RMS error should be considered preferably to the mean error, since the hip frequently underestimates or overestimates the CM due to differences in swimmers’ technique (negative errors cancelled by the positive ones), and because RMS is considered a conservative estimate of accuracy (Allard et al., 1995).

Furthermore, high and very high positive correlation coefficients were found between the hip and the CM regarding horizontal swimming velocity and displacement, as seen in front crawl (Costill et al., 1987; Maglischo et al., 1987, Figueiredo et al., 2009), backstroke (Maglischo et al., 1987), breaststroke (Costill et al., 1987; Maglischo et al., 1987), and butterfly (Maglischo et al., 1987; Barbosa et al., 2003). Considering each swimmer individually, a positive correlation was observed between the hip and CM values regarding velocity (ranging from 0.50 to 0.83), which is in accordance with Maglischo et al. (1987) in front crawl technique (values between 0.86 and 0.96, with a mean coefficient of 0.87). These data, associated with the obtained high digitize-redigitize reliability values, evidence that, although there is an associated error that should be taken into account, the hip reflects satisfactorily the CM motion in front crawl when swimming at moderate intensity.

The velocity to time curve obtained for one swimmer for both CM and hip showed similar patterns of positive and negative accelerations as described in the literature (Maglischo et al., 1987; Craig et al., 2006): both CM and hip decelerated during the downsweep phases (that are coincident with the recovery of the opposite arm) and in the transition from one propulsive phase to another, and both body points accelerated during the catch, insweep and upsweep phases. Thus, coaches should incorporate specific training drills aiming to perform faster transitions between propulsive phases, as well as to finish the stroke at maximal arm velocity. It was also evident that swimmers choose a catch-up inter-arm coordination mode that is typical of moderate paces due to a long gliding phase (Schnitzler et al., 2008; Seifert and Chollet, 2009; Seifert et al., 2010). In fact, the existence of a discontinuity between the end of the propulsion of one arm and the beginning of propulsion of the other arm is typical of front crawl swimming at moderate intensities (Seifert and Chollet, 2009; Seifert et al., 2010). Thus, coaches should not advise swimmers to adopt superposition arm synchronization when implementing aerobic pace training series. Furthermore, it was also evidenced that the hip presents higher and lower forward velocity peaks magnitude compared to CM, as shown by Maglischo et al. (1987) for higher swimming intensities.

Notwithstanding that the forward velocity and displacement of the hip and CM are similar, and the evidence that the IVV determination using the hip is reliable, allows multiple cycles to be evaluated and enables the assessment of fatigue (Holmér, 1979; Maglischo et al., 1987), differences between hip and CM were found for the IVV. Such differences corroborates the literature (Figueiredo et al., 2009), and might be explained by the inter-segmental actions during the front crawl swimming cycle that frequently changes the CM position (Barbosa et al., 2003). In addition, the CM v_max_ and v_min_ values seem to be over and underestimated (respectively) by the hip values, as previously proposed by Psycharakis and Sanders (2009). In fact, when the arms in front crawl accelerate the body mass, they simultaneously move backwards with respect to a body fix landmark refraining the acceleration of the CM. The same is expected in case of the negative accelerations determined by body drag prevalence: during the drag dominated phases, one arm is recovering (moving forward with respect to a fix body point), reducing the total negative acceleration of the CM comparatively to a body landmark. Meanwhile, the observed differences may be lower than previously found in maximal front crawl swimming (Maglischo et al., 1987; Figueiredo et al., 2009; Psycharakis and Sanders, 2009), because the current study was conducted at moderate intensity that is also characterized by lower positive and negative intracyclic accelerations (Tella et al., 2008). In fact, increases in IVV are associated with the swimmers acceleration capacity that is greater at higher swimming intensities (Schnitzler et al., 2010).

Despite the dissimilarities, the kinematics of the hip and CM are easily explainable. It should be emphasized that errors associated with the CM assessment, particularly concerning images quality, digitizing, calibration, refraction and reconstruction, as well as inertial models (Vilas-Boas et al., 2010; Figueiredo et al., 2011), may also contribute to the observed differences. In addition, the CM position varies (e.g. with the breathing pattern and with the distribution of body fluids; Bartlett, 2007), what results in less accurate estimation when shoulder movement is involved (Plagenhoef, 1976), as it occurs in front crawl swimming. Finally, no differences were observed between the CM and hip concerning the timing of v_max_ and v_min_, despite the high RMS values, as reported by Psycharakis and Sanders (2009). Furthermore, despite that the calculation of hip velocity based on 2D analysis may increase the possibility of errors, lower and/or similar RMS values were registered for the above-referred velocity variables than those reported using a 3D approach (Psycharakis and Sanders, 2009).

The current results showed that IVV assessed from the hip could be useful to characterize swimming technique, evidencing the combination between propulsive and resistive forces. Our data suggests that, when implementing aerobic conditioning training in swimming, coaches should include drills aiming to accomplish faster transitions between propulsive phases, and to finish the front crawl stroke at maximal arm velocity. It is evidenced that plotting the hip to assess swimmer’s forward velocity and displacement is a simple and fast process that enables evaluating multiple cycles and giving quick feedback to swimmers. However, when using the hip as a measure of forward velocity and/or displacement, the associated error (∼7 and 3%) should always be taken into consideration.

## Figures and Tables

**Table 1:** Mean ± SD values of velocity and displacement of the centre of mass and hip, and the mean error, root mean square (RMS) error and percentage error. Mean intraclass correlation coefficient (ICC) between hip and centre of mass is also displayed (n=16)

Variable	Centre of mass	Hip	Mean error	RMS error	Percentage error	Mean ICC	Significance (p)
velocity (m/s)	1.06±0.26	1.06±0.32	0.00	0.18	7.54	0.71	p<0.001
displacement (m)	2.16±0.32	2.16±0.34	0.06	0.07	3.24	0.99	p<0.001

**Table 2:** Mean ± SD values of the centre of mass and hip velocity related variables (p value is also shown). The mean and RMS errors are also displayed (n=16)

Variable	Centre of mass	Hip	Paired samples t-test (p)	Mean error	RMS error
IVV	0.19±0.05	0.25±0.08	< 0.001	−0.06	0.07
v_max_ (m/s)	1.59±0.27	1.73±0.29	0.001	−0.14	0.19
v_min_ (m/s)	0.57±0.22	0.46±0.21	0.003	0.11	0.17
Relative v_max_ (%)	147.77±0.18	159.91±0.17	0.002	−12.14	17.37
Relative v_min_ (%)	53.03±0.20	43.22±0.21	0.003	9.81	14.45
Timing v_max_ (%)	35.11±0.26	45.44±0.26	0.257	−10.33	35.30
Timing v_min_ (%)	48.17±0.24	55.51±0.25	0.171	−7.34	21.15

*IVV: intracycle velocity variation, v_max_: maximum velocity, v_min_: minimum velocity, and RMS: root mean square*
